# Organizational development trajectory of a large academic radiotherapy department set up similarly to a prospective clinical trial: the MAASTRO experience

**DOI:** 10.1259/bjr.20140559

**Published:** 2015-03-09

**Authors:** M Jacobs, L Boersma, A Dekker, E Hermanns, R Houben, M Govers, F van Merode, P Lambin

**Affiliations:** ^1^ Department of Radiation Oncology (MAASTRO), School for Public Health and Primary Care-Health Services Research, Maastricht University Medical Centre (MUMC+), Maastricht, Netherlands; ^2^ Department of Radiation Oncology (MAASTRO), GROW-School for Oncology and Developmental Biology, Maastricht University Medical Centre (MUMC+), Maastricht, Netherlands; ^3^ Department of Health Services Research, School for Public Health and Primary Care (CAPHRI), Maastricht University, Maastricht, Netherlands; ^4^ Executive Board Maastricht University Medical Centre (MUMC+), Maastricht University, Maastricht, Netherlands

## Abstract

**Objective:**

To simultaneously improve patient care processes and clinical research activities by starting a hypothesis-driven reorganization trajectory mimicking the rigorous methodology of a prospective clinical trial.

**Methods:**

The design of this reorganization trajectory was based on the model of a prospective trial. It consisted of (1) listing problems and analysing their potential causes, (2) defining interventions, (3) defining end points and (4) measuring the effect of the interventions (*i.e.* at baseline and after 1 and 2 years). The primary end point for patient care was the number of organizational root causes of incidents/near incidents; for clinical research, it was the number of patients in trials. There were several secondary end points. We analysed the data using two sample *z*-tests, *χ*^2^ test, a Mann–Whitney *U* test and the one-way analysis of variance with Bonferroni correction.

**Results:**

The number of organizational root causes was reduced by 27% (*p* < 0.001). There was no effect on the percentage of patients included in trials.

**Conclusion:**

The reorganizational trajectory was successful for the primary end point of patient care and had no effect on clinical research. Some confounding events hampered our ability to draw strong conclusions. Nevertheless, the transparency of this approach can give medical professionals more confidence in moving forward with other organizational changes in the same way.

**Advances in knowledge:**

This article is novel because managerial interventions were set up similarly to a prospective clinical trial. This study is the first of its kind in radiotherapy, and this approach can contribute to discussions about the effectiveness of managerial interventions.

Medical professionals are naturally sceptical about management theories that are usually supported by a much weaker level of evidence than are evidence-based medicine Levels I–II. Research on organizational design and before and after study designs frequently lacks quantitative measures and has limited scientific evidence.^1^ Approaching management problems similarly to prospective clinical trials could narrow the potential gap between physicians and managers in solving managerial issues and consequently enhance managerial outcome performance.

The central management challenge of the radiotherapy institute MAASTRO Clinic reported in this study is the combination of lean patient care processes, growth, innovation and clinical research activities (which are, by definition, highly variable). This is not a unique challenge; instead, it is a manifestation of organizational ambidexterity. Ambidexterity refers to an organization's ability to simultaneously engage in sufficient exploitation (*i.e.* efficiently manage current operations) to ensure its current viability and, simultaneously, to devote enough energy to exploration (*e.g.* of new technologies, treatments, markets, organizational resources) to ensure its future viability.^2^

More specifically, exploitation is not only about efficiency but also about productivity, control, certainty and variance reduction. Exploration concerns research, discovery, autonomy, innovation and embracing variation.^2^

Exploitation and exploration have often been seen as a trade-off.^2–4^ March^5^ stated in his pioneering article that the two are fundamentally incompatible. Organizations pursuing both strategies simultaneously will become stuck in the middle. Since the publication of this paper, more and more papers have been published about ambidextrous organizations strategies. These studies suggest that under certain well-specified circumstances, related to leadership and organizational design, it may be possible for organizations to simultaneously excel in both exploration (*e.g.* research) and exploitation (*e.g.* innovation implementation and daily operation).^6^ However, no quantitative research has yet verified this statement. This study aims to test our hypothesis that a reorganization trajectory that deals with the above-mentioned management challenge and is set up similarly to a prospective clinical trial will improve both patient care processes and the output of clinical research activities.

## METHODS AND MATERIALS

### Design

We started the reorganization trajectory with a multidisciplinary working group that consisted of radiation oncologists, medical physicists, radiation technologists, administrators and representatives of the employee council, supervised by a consultant (professor in organizational design). It was based on a protocol defining an *a priori* testable hypothesis with specific, measurable, attainable, realistic, time-bound indicators. The approach of this reorganization trajectory was based on the model of a prospective trial. The multidisciplinary working group applied the following steps: (1) listing problems and analysing their potential causes, (2) defining interventions and (3) defining end points to measure the effect of the interventions, both at baseline and at 1 and 2 years after the start of the trajectory.

The interventions (changes in the organization) were approved by the management board, the medical and physics staff, the employee council, the patient council and the board of supervisors. We did not change anything in the functional tasks of the radiation technologists; in our department, all radiation technologists are versatile, meaning that they perform scans (positron emission tomography and CT) and make treatment plans, and work at the linear accelerator (linac). To limit the number of skills required for their roles, radiation technologists are specialized in two to four disease entities.

### Measures

The multidisciplinary working group devised several end points to measure the effect of the reorganization trajectory at baseline, and at 1 and 2 years after the start of the trajectory (see the Results section). These end points were related to either patient care or clinical research and were measured in the following ways.

The main patient care end point was the number of organizational root causes of incidents/near incidents. We used the existing incident reporting system to quantify root causes. In this system, all incidents are measured and classified according to their root cause^7–10^ (for the potential types of root causes in this system, see Supplementary material). A near incident is an unforeseen event in the process that did not reach the patient. If this disturbance actually reaches the patient, it is called an incident.

The secondary patient care end points were measured in different ways. Successfully accomplished and approved policy projects were measured as a percentage of all approved projects in the policy plan. Job satisfaction was measured using an internationally validated questionnaire, the Index of Work Satisfaction.^11,12^ Labour productivity was measured as reimbursement per full-time equivalent (FTE). In the Netherlands, the reimbursement per treatment depends on the labour intensity of the treatment and reflects the weighted number of treatments. Teamwork was measured across and within units according to the Hospital Survey on Patient Safety Culture conducted by the Agency for Healthcare Research and Quality.^13,14^ Variations in patient waiting times between work units were measured using our logistics database: waiting time was defined as the time between registration and the first fraction of radiotherapy.

We also decided to measure some other patient care parameters because these could be unintentionally influenced by the reorganization. Patient satisfaction was measured by our own green/yellow/red card system in which patients give us a compliment or a moderate or strong complaint. Furthermore, waiting time was measured using our logistics database and compared with the standards of the Dutch Society for Radiation and Oncology (80% of patients must start their treatment within 21 days after referral and 100% within 28 days).

The main clinical research end point was the number of patients in trials. This was measured by the ratio between the total number of patients seen in a year and the proportion of those patients in trials (not including patients in biobank studies).

Finally, we performed a qualitative evaluation at 1 and 2 years after the start of the trajectory. In each functional group, a meeting was organized to ask professionals how they felt about the reorganization trajectory.

### Statistical analysis

In 2010, 3961 treatments were carried out in 3019 patients. In the Netherlands, radiation treatment of every single target volume is counted as one treatment, so a new patient can have more treatments. In the process of delivering these treatments, we identified 2184 organizational root causes of incidents/near incidents (*i.e.* this is 55% when we normalize this figure to the number of treatments; see the Discussion section for further consideration). Assuming that these figures would be representative for the baseline values for the primary end point of patient care, we hypothesized that a 20% reduction should be obtained (*i.e.* from 55% to 44%). Assuming at least 4000 treatments are delivered per year and given an *α* level of 0.05, the power of the analysis to detect a difference of 20% is >0.99. For the primary end point of clinical research, we assumed a baseline value of 5% of patients included in clinical trials (excluding patients participating in the biobank) over a total of 3000 patients, which allowed us to detect an increase of 34% (*i.e.* to 6.7%) with a power of 0.80 and an *α* of 0.05.

We used various methods to measure different variables. We used *χ*^2^ tests to analyse the number of organizational root causes of incidents/near incidents, the number of patients in trials, teamwork within units, teamwork across units, the number of completed projects and patient satisfaction. We used an analysis of variance to analyse technologists' average job satisfaction and used a *post hoc* Bonferroni correction to measure changes in average job satisfaction over the years. A Mann–Whitney *U* test was used to analyse differences in waiting times between units and a *z*-test for proportions for the changes in the percentages of patients reaching the waiting standards. In all cases, we assumed a significance level of 0.05.

## RESULTS

### Listing problems and root causes

**Figure 1.**
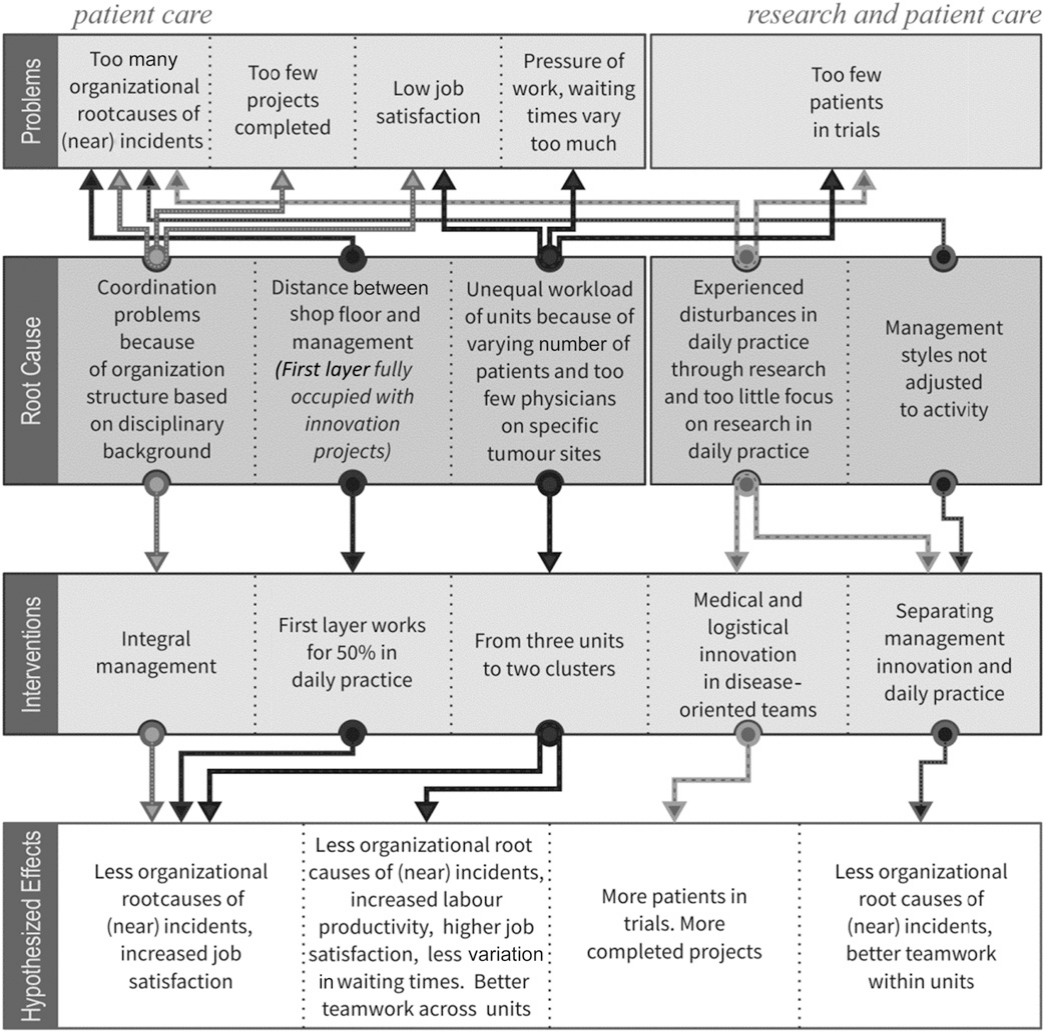
Overview of problems, root causes, interventions and hypothesized effects.

The following problems related to patient care were listed by the multidisciplinary working group that consisted of radiation oncologists, medical physicists, radiation technologists, administrators and representatives of the employee council (Figure 1, first row):

• too many organizational root causes of incidents/near incidents in our patient processes, resulting in disturbances requiring improvisation and rework
• many incomplete care innovation projects (<50%)
• job satisfaction of radiation technologists was too low (4 on a scale of 0–7)
• differences in work pressure from one working unit to other units
• problems related to the combination of patient care and research, mainly resulting in too few patients being included in trials (5.1%).

The group discussed root causes and, to a large extent based on literature, assumed that there were five root causes of our problems (Figure 1, second row):

• co-ordination problems and process breakdown problems caused by our functional organizational structure being based on professional disciplines instead of on patient processes^15–18^
• distance between front-line employees and management^2,6,18^
• disturbances of research on daily practice, and too little focus on the importance of research in the clinic^3,19,20^
• lack of adjustment of management styles to activity, more specifically to daily operations, research and innovation adoption^20–23^
• an unequal workload between the three existing work units [each unit treated only a specific type of tumour site: Unit 1 (genitourological/gastroenterology tumours); Unit 2 (thoracic/lymphoma/breast tumours); Unit 3 (head and neck/neurology/children/sarcomas/breast tumours)].

### Determining interventions and end points

**Figure 2.**
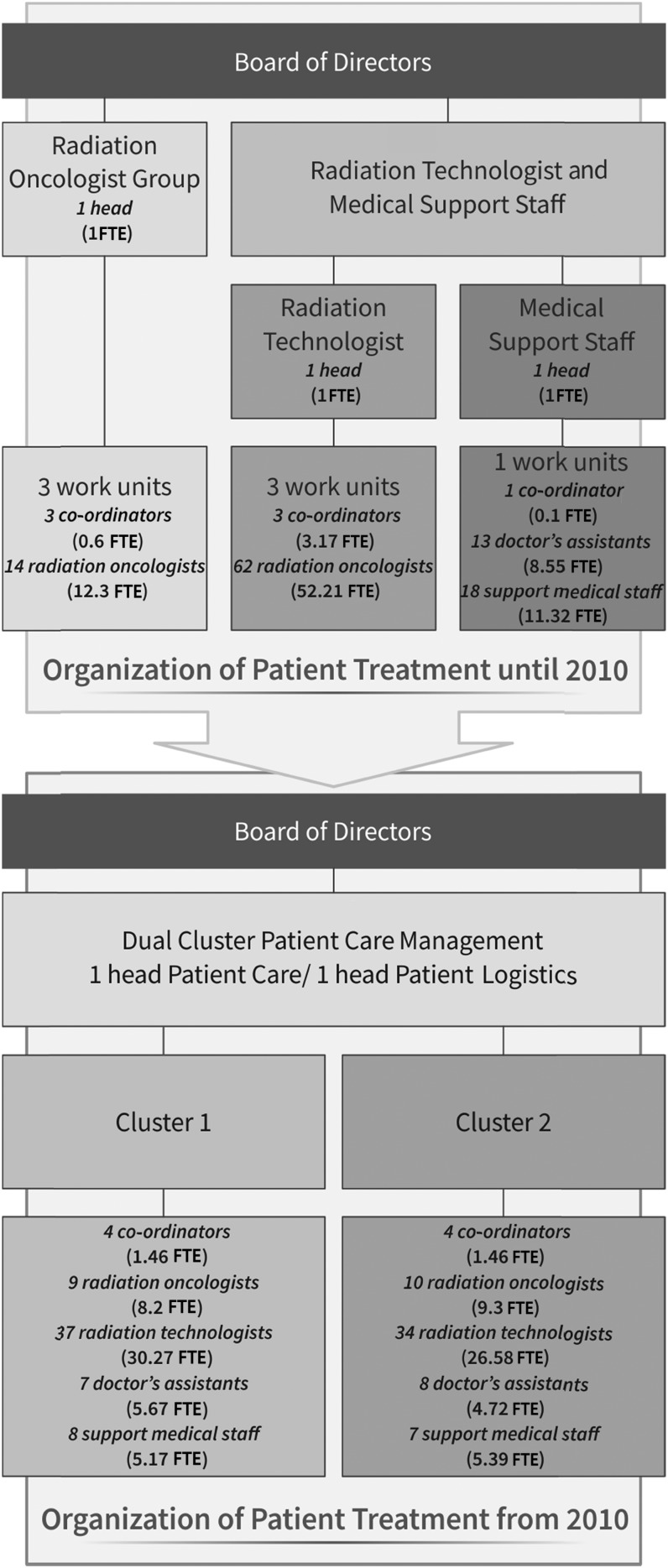
Transition of the organizational structure. FTE, full-time equivalent.

Under the consultant's supervision, the group devised several interventions (Figure 1, third row):

• Moving from three independent work units to two clusters: patients with an oncological at disease above the diaphragm were treated in one cluster and patients with disease below the diaphragm were treated in the other cluster. Two patient groups (patients with palliative care and patients with breast cancer) were treated in both clusters to level the workload between them. This is possible because in the Netherlands, palliative care is not a specialization for radiation oncologists; since palliative patients comprise approximately 50% of all patients, all radiation oncologists frequently apply palliative radiotherapy. For breast cancer, dedicated radiation oncologists and technicians are present in both clusters.
• Changes to management teams:
   ◦ Introduction of a multidisciplinary co-ordination team (*i.e.* including a radiation oncologist, radiation technologist and logistic administrative person) for each cluster; in the previous setting, each functional group had its own leader. The span of control (number of employees supervised) changed, as shown in Figure 2; in the old situation, each unit had a co-ordinator for radiation oncologists (supervising 7 radiation oncologists) and 1 for radiation technologists (supervising 20 technicians). For the administrative/supporting employees, 1 manager was assisted by a co-ordinating doctor's assistant 4 h a week and together they supervised 31 people. In the new situation, the span of control increased by 50% for the co-ordinators of the physicians and technologists and decreased by 50% for the supervisors of the administrative/supporting staff.
   ◦ The co-ordinators were required to work in daily patient care for at least 50% of their working hours. Before the reorganization, this was already the case for the co-ordinators of the radiation oncologists but not for the co-ordinators of the technologists. They did not work in daily practice at all. For the administrative/supporting staff, two co-ordinators were appointed, both of which were asked to work 50% in daily practice.
   ◦ Introduction of an integral management quartet supervising the co-ordination teams: one pair for daily patient care (*i.e.* a radiation oncologist and a logistics manager) and one pair for innovation (*i.e.* a medical physicist and a manager of innovation).
• Apart from the 2 clusters, where daily operational practice was carried out, we introduced 11 multidisciplinary disease-oriented teams (*e.g.* the lung team, the head and neck team), each consisting of 13–23 employees. The major task for these teams was to maintain and improve treatment protocols, develop innovation and develop a yearly policy plan for a specific disease site. In this step of the reorganization trajectory, it was not considered feasible to give these teams daily operational responsibilities, since many professionals were members of more than one team. Therefore, our organizational design did not completely change from functional to process based (for the organization chart of daily operation, see Figure 2).

Finally, the group devised several end points (Figure 1, fourth row) to measure the effect of the reorganization trajectory.

### Results of the measures

The primary end point for patient care, organizational root causes of disturbances in patient processes, improved by 27% in 2 years (*p* = 0.016) from a 55% occurrence in all treatments to 40% of all treatments. This was a 4% improvement over the target obtained in the sample size calculation. Two secondary end points, job satisfaction (*p* = 0.004) and teamwork across units (*p* ≤ 0.001), improved as well. Satisfaction of technologists improved from 5.7 to 6.4, converted to a scale of 0–10 (*p* < 0.001). The following components improved significantly: task requirements (from 5.3 to 6.2; *p* = 0.032), autonomy (from 5.4 to 6.0; *p* = 0.035), impact administration (from 3.8 to 4.7; *p* < 0.001) and pay (from 4.3 to 5.7; *p* < 0.007). Also, the difference in waiting time between clusters improved from 4.3 to 2.2 days (*p* = 0.022) (Table 1).

**Table 1.** End points

End points	T0, baseline	T1, Year 1	T2, Year 2	T0–T1, *p*-value	T0–T2, *p*-value	T1–T2, *p*-value	*χ*^2^ test
Primary end points							
Number of reported organizational root causes of incidents/near incidents/total number of treatments	2184/3961 (55.1%)	1803/3802 (47.4%)	1588/3939 (40.3%)				**<0.001**
Number of organizational root causes of incidents/near incidents without linear accelerator reports/total number of treatments	1399/3961 (35.3%)	1110/3802 (29.2%)	1062/3939 (27.0%)				**<0.001**
Number of patients in trial	153/3019 (5.07%)	158/3015 (5.24%)	175/3124 (5.60%)				0.634
Secondary end points							
Waiting time							
Percentage of patients treated within 21 days	81.8	79.3	62.6		**<0.001**	**<0.001**	
Percentage of patients treated within 28 days	93.7	92.9	84.3		**<0.001**	**<0.001**	
Median differences in waiting time between units	4.26	1.75	2.23	**≤0.001**	**0.022**		
Teamwork across units (percentage of scores reported as positive)	34		51				**<0.001**
Number of completed projects (implementation yes/no)	18/19 (48.6%)	23/20 (53.5%)	26/11 (70.3%)				0.140
Average job satisfaction technologists on a scale of 0–7 (±standard deviation)	4.04 (±0.63)	4.16 (±0.56)	4.42 (±0.59)		**<0.001**	**0.004**	
Number of technologists	47	43	50				
Treatment reimbursement in (Euro/full-time equivalent)	188.000	180.000	184.000				
After correcting for training, new equipment and software			188.5				
Patient satisfaction							
Number of complaints	75/3019	96/3015	101/3124				**0.006**
Number of compliments	135/3019	107/3015	165/3124				
No response	2809/3019	2812/3015	2858/3124				
Excluding the new satellite introduced in 2012							
Number of complaints			83/2782				0.204
Number of compliments			113/2782				
No response			2586/2782				
Teamwork within units (percentage of scores reported as positive)	70		71				0.930

Values in bold indicate statistically significant values.

Not all of the end points changed for the better. Reimbursement per FTE appeared to deteriorate. However, when we corrected this end point for FTEs devoted to training on new accelerators and new software (see the Discussion section), we found a slight increase in labour productivity.

One of the two parameters that we measured to study the possible unintentional influences of our reorganization changed. Patient waiting time worsened for both the 21-day standard and the 28-day standard (*p* ≤ 0.001). Patient satisfaction showed no change.

The number of completed projects and teamwork within units were also unchanged. The primary end point for research, the number of patients in trials, also did not change significantly.

The qualitative evaluation demonstrated that the groups that were performing well or were strongly connected beforehand were less satisfied with the changes than the other groups.

## DISCUSSION

This study shows that (a) a reorganization trajectory set up similarly to a prospective clinical trial is possible and (b) 2 years after the start of a hypothesis-driven reorganization trajectory, the main end point for clinical patient care (*i.e.* organizational root causes of incidents/near incidents) improved by 27% (*p* = 0.016). The main end point for clinical research (*i.e.* the number of patients in trials) did not change significantly. Since several other changes occurred in the department during these 2 years, it is difficult to draw firm conclusions about the causal relationship between the reorganization trajectory and end points. For instance, two major processes that are likely to have influenced our end points are (1) in 2012, we set up a satellite department that required an increased speed (*e.g.* of digitalization) and (2) in 2011 and 2012, we replaced our six linacs and the treatment planning system. Nevertheless, we can conclude that despite these major confounding events, most of the end points, including one main end point, improved and only one end point worsened.

### Primary end point: reduction of organizational root causes of incidents/near incidents

**Figure 3.**
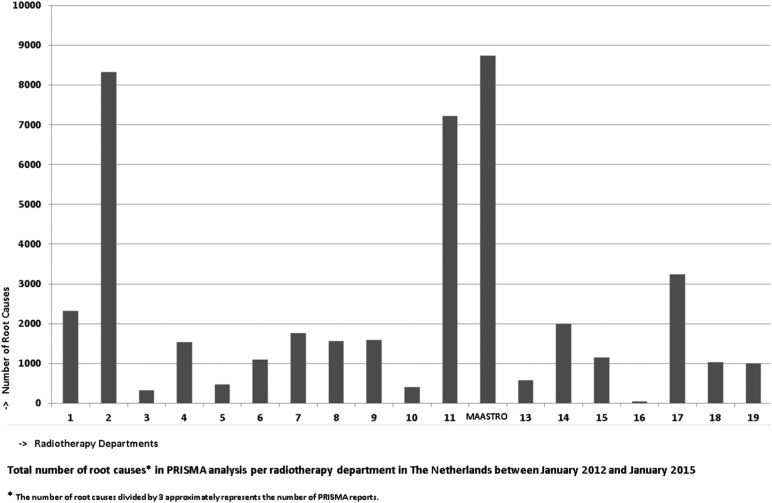
Number of safety system reports in the Netherlands. The clinic in this study is number 7857. The horizontal axis shows the different types of incidents/near incidents; the vertical axis shows the number of reports.

The number of organizational root causes of incidents/near incidents before the intervention seems quite high and could give the impression that this allows an obvious improvement with minimal effort. This needs to be clarified. Many incidents/near incidents is not, by definition, a representation of a poorly functioning organization. On the contrary, it often reflects the willingness of the clinic to report incidents/near incidents.^24,25^ In another study, we concluded that our clinic has a long history with safety improvement, which explains the high levels of willingness to report incidents/near incidents^26^ (for the number of reports per radiotherapy centre in the Netherlands, see Figure 3). Furthermore, the number of real incidents in this clinic was low (*e.g.* in 2012, 98% of all incidents did not reach patients at all). We related the number of incidents/near incidents to the number of treatments as a normalization factor; this does not mean that 55% of the treatments had problems.

Before the reorganization trajectory, our institute had a functional organizational structure with different departments for radiation oncologists, technologists, physicists and supporting staff. Literature^15–18^ has shown that this organizational structure results in a very complex system of flows and queues with many transfer points from one department to another that result in a long, slow and often inaccurate course. Defects in the transfer points result in an exponential increase of the effects and costs in the chain. In such a functionally organized structure, the organization is divided on the basis of the disciplinary background of the staff. Every discipline tries to optimize its own function instead of the whole patient process. As a result, processes frequently break down, resulting in co-ordination that becomes not only very important^17^ but also complex.^16,17,27^

Based on these premises in the literature, we assumed that a less functional organizational design would result in fewer incidents/near incidents with organizational root causes. In preceding years, several lean projects to improve compliance and quality in radiotherapy treatment were accomplished, but they all exclusively focussed on patient processes and not on organizational design. After an intervention on organizational design, further improvements were expected.

The reduction of organizational root causes of incidents/near incidents did occur, and we decided to investigate the causes. Some employees reported that the replacement of all the linacs and software meant that there were fewer technical-related organizational problems and concluded that the organizational intervention might not have caused the reduction of incidents/near incidents with organizational root causes. We therefore looked specifically at incidents/near incidents, excluding linac issues. Here, we also found fewer reported events in proportion to the total number of treatments (*p* ≤ 0.001) and interventions (Table 1).

Another explanation could be a decreased willingness to report incidents/near incidents. However, a further safety study at the MAASTRO clinic (Maastricht, Netherlands) found, based on a triangulation of methodologies (two surveys were distributed three times, workshops were performed twice, data from an incident reporting system was monitored and results were explored using structured interviews with professionals) that the decreased number of reported incidents/near incidents was not explained by decreased safety awareness or a decreased willingness to report, but by improved treatment processes.^26^

### Primary end point: increase of patients in trials

At the start of our reorganizational trajectory, we included 5% of our patients in clinical trials. This is much lower than participation rates reported in the UK,^28^ but 5% is considered to be a reasonable score in a radiotherapy department in continental Europe or the USA.^28,29^ The number of patients in trials did not increase after the start of our reorganizational trajectory.

Increasing participation rates for clinical therapeutic trials is a complex matter; participation rates depend on (a) key structural barriers (*e.g.* lack of physician's recommendation to participate or limited availability of active protocols), (b) personal-related barriers (*e.g.* lack of knowledge, fear of receiving the placebo) and (c) emotional barriers on the part of patients.^30^ The trial design can be another barrier.^31^ We did not completely fulfil the conditions necessary to accomplish an ambidextrous strategy as described in literature. A lot more work beyond just organizational design is required to build on this study.^5,32–34^ Therefore, it is not surprising that changes to only the organizational design had negligible effects with respect to this end point.

### Management interventions

**Figure 4.**
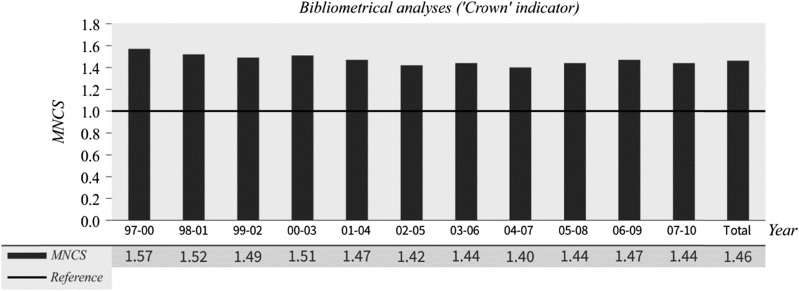
“Crown” indicator. MNCS, mean normalized citation score.

**Figure 5.**
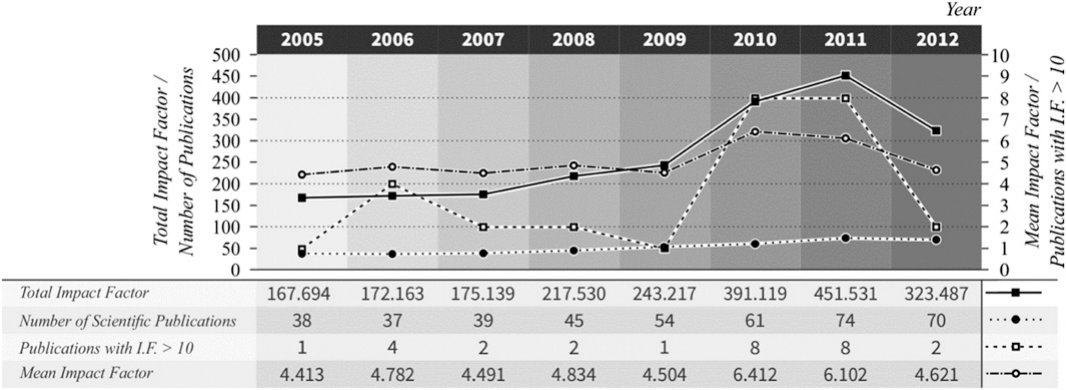
Publication and impact factors. I.F., impact factor.

Our organization is carrying out research, as is clear from the number of publications and the Crown indicator (a well-known bibliometric indicator of research performance calculated from the number of citations compared with the expectation value for the scientific field: a value >1.2 indicates a high scientific impact; Figures 4 and 5).

From the literature, we know that research as an innovation-generating process is facilitated in different organizational conditions (*e.g.* culture, the degree of control) as innovation implementation^19^ and daily operations.^3,4,35^ The different processes also have consequences for the leadership task.^20–23^ Research is closely linked to exploration and, sometimes also, requires thinking outside the box and going beyond routines or common assumptions and experimentation. Innovation implementation and daily operations are closely linked to exploitation. They also need the above-mentioned research-related activities to some extent but are characterized more by efficiency, goal orientation and routine execution. These activities are not completely unnecessary for research but undoubtedly play second fiddle.^33^ When exploration is needed, leaders need to behave in a way that encourages doing things differently and experimenting, making room for independent thinking, and acting and supporting attempts to challenge established approaches. Exploitation requires leaders to take corrective actions, set specific guidelines, establish routines, and monitor goal achievement and plans. Doing all these activities requires leaders to balance exploration and exploitation, to integrate them when needed and to flexibly switch between them as the situation requires.^33^

Based on the findings above, we assumed that some of the listed problems were caused by the fact that MAASTRO supervisors usually used just one style (some mainly explorative, others mainly exploitative) regardless of whether they were involved in research, daily operations or innovation implementation. On this basis, we hypothesized that the organizational changes to management, especially distinguishing between innovation and daily operation, would also contribute to fewer incidents/near incidents with organizational root causes and the improvement of some other end points.

This was also expected from the other changes in the management structure. Less hierarchical levels and integral management were expected to benefit work outcome, and this was indeed the case as we looked to root causes of organizational incidents/near incidents categorized as management decisions. This is described in our report system as errors in patient processes as a result of managerial decisions. These decisions are often taken by management when employees experience conflicting demands and need management to tell them what to do or when time pressure requires management to prioritize activities. Organizational root causes of incidents/near incidents referring to organizational management decisions decreased by 42% (*p* = 0.002). We think this is another indicator for the success of our intervention.

Although we discussed whether it was good or bad to increase the span of control, and the literature often states that a “feasible span of control” is needed,^36^ our increased span of control did not hamper the decrease of incidents/near incidents. Nevertheless, there may be room for further improvement on this issue in the next step of our reorganizational trajectory.

We also expected that job satisfaction would increase because employees would experience more attention and less distance. This was only the case for technologists, who had the lowest levels of satisfaction before the intervention. Increasing job satisfaction more radically obviously requires more interventions, as we will describe in the section From three units to two work units.

### Introduction of disease-oriented teams

Implementing multidisciplinary teams dedicated to a specific patient group had the intended purpose of mobilizing mental power to innovate on the treatment itself and on logistics. We hypothesized that this intervention would improve both primary end points. We also expected to see beneficial effects on completed projects and teamwork within units.

The reduction of organizational root causes of incidents/near incidents occurred, which can only partly be ascribed to better multidisciplinary teamwork. This relationship, however, is in line with prior findings about the strength of lateral relationships (not only following the hierarchical way). These lateral relationships make it possible to increase the capacity to process information, which is needed to fulfil all patient treatment tasks for all employees involved in a certain treatment.^27,37,38^

We did not see an increase in the percentage of completed projects, which can be ascribed to a lack of supervision. We discovered in the qualitative evaluation that for a team to function well, it was more important for the chair to have leadership skills than to be a radiation oncologist. In the management team, we are now creating conditions to improve self-management of the teams with a balance between central control and local autonomy.^18^

Teamwork within units did not improve either. The qualitative evaluation showed that people saw the teams in daily operation as their unit and not the disease-oriented team, which in fact is only a meeting setting where people reflect together on improvements in daily operations.

### From three to two work units

The decision to move from three to two work units aimed to improve productivity because dividing the workload became easier. There was less variation in waiting times between units, which is an indicator that work pressure is more equal in the new situation (*p* ≤ 0.022). This is unlikely to have been affected by the new satellite or the new machinery.

As a result, we also expected to see improvements on job satisfaction and teamwork across units. Labour productivity did not increase as measured by the treatment reimbursement/FTE, but we think this end point was heavily affected by the introduction of the new machinery. For example, the technologists were required to attend 1500 4-h training shifts on top of normal production. Correcting for this number of FTEs, we found an increase in labour productivity to €188.5 thousand per FTE, which is €500 per FTE above the 2010 level.

The change from three to two working units implied that the radiation technologists had to broaden their skills, since a greater variety of tumours was treated in the new cluster than in the old work units. In the qualitative evaluation, discussions began about whether specialization^39^ or generalization was the best course. The Dutch Society for Radiation and Oncology has made a clear statement that it is necessary for radiation oncologists to specialize in two to four diseases, whereas we were heading in the opposite direction for the technologists. The radiation oncologists were especially worried that this would lead to poorer quality, since they assumed a relationship between quality and volume,^40^ although no research has been carried out on this relationship in radiotherapy.^41^

Another disadvantage of the large clusters was that the work became more anonymous. An individual did not always know who was working in the same patient process in the preceding or following steps. In the qualitative evaluation, this was most often mentioned by teams who were close and/or performing well before the reorganization. Despite the fact that teamwork within units did not worsen in the quantitative evaluation, this is a point of particular interest.

The construction of teams with specific characteristics is essential for employee involvement,^18^ which is an important determinant of job quality and job satisfaction.^42^ These characteristics are complete area of responsibility with as little division of labour as possible, sufficient regulatory authority, insight and information, and accountability with efficient feedback mechanisms.^18^ The reorganization did not result in teamwork with the above characteristics, especially because the task design was not based on a complete series of interrelated activities. Therefore, it is not surprising that job satisfaction only slightly improved for radiation technologists and not at all for other employees.

On the basis of the above insights, we recently decided to take the next step in our journey from a functionally based organizational design towards a process-based one. We will establish more process-based teams in daily operation with more specialization as well as the above-described team characteristics.

### End points that could unintentionally be influenced by the reorganization

#### Waiting time

In the Netherlands, a standard set by the Dutch Society for Radiation and Oncology states that 80% of all patients must have their first treatment within 21 days after referral and 100% within 28 days. The standard is not differentiated by disease. In 2010, we achieved the 21-day standard with a value of 81.8%. From 2010 to 2011, no significant change took place (the percentage was 79.3% in 2011). In 2012, the year in which MAASTRO opened the satellite and replaced five linacs, the percentage significantly reduced to 62.6% (*p* < 0.001). In 2010, we failed to meet the 28-day standard (only 93.7% of patients had their first treatment within 28 days). In 2011, this percentage dropped to 92.2%, which is not a significant change. In 2012, the percentage significantly reduced to 84.3% (*p* < 0.001). Because there was less linac capacity available during the replacement and there was also less staff available (employees received 6000 h of training and also had to settle in to the new satellite), there is probably a relationship between the increased waiting time and the confounding events. We found no connection with the reorganization.

#### Patient satisfaction

We observed no influence of the reorganization. Patient satisfaction, excluding the satellite, did not change significantly (including the satellite, there was a perceptible improvement).

### The ambidextrous challenge of our study

The ambidextrous challenge to simultaneously improve lean efficient patient care processes, growth and innovative (per definition, highly variable) clinical research activities with our reorganization trajectory was not completely successful within the 2 years after the reorganization.

Also, in the literature, it is stated that organizational design and structure alone are not enough to reach sustainable change and performance improvement in complex situations.^4,18^ It may have been possible to improve both end points simultaneously if we had paid more managerial attention to the barriers for clinical trial participation and had also more closely followed the actions suggested in literature on ambidexterity. However, we think, in that case, the changing of the organization would have been more far reaching. We believe that combined with the two other major interfering processes (replacement of the linacs and the start of the satellite), this would have been a too-drastic change with accompanying risks. In future research, we will study the effect of additional actions in order to improve both end points.

We think that other radiotherapy centres (academic and otherwise) that want to improve patient care processes and research participation must decide if they want to do it simultaneously or successively. Our study showed that doing it simultaneously requires a plan that goes beyond organizational design.

### The set-up of the reorganization

**Figure 6.**
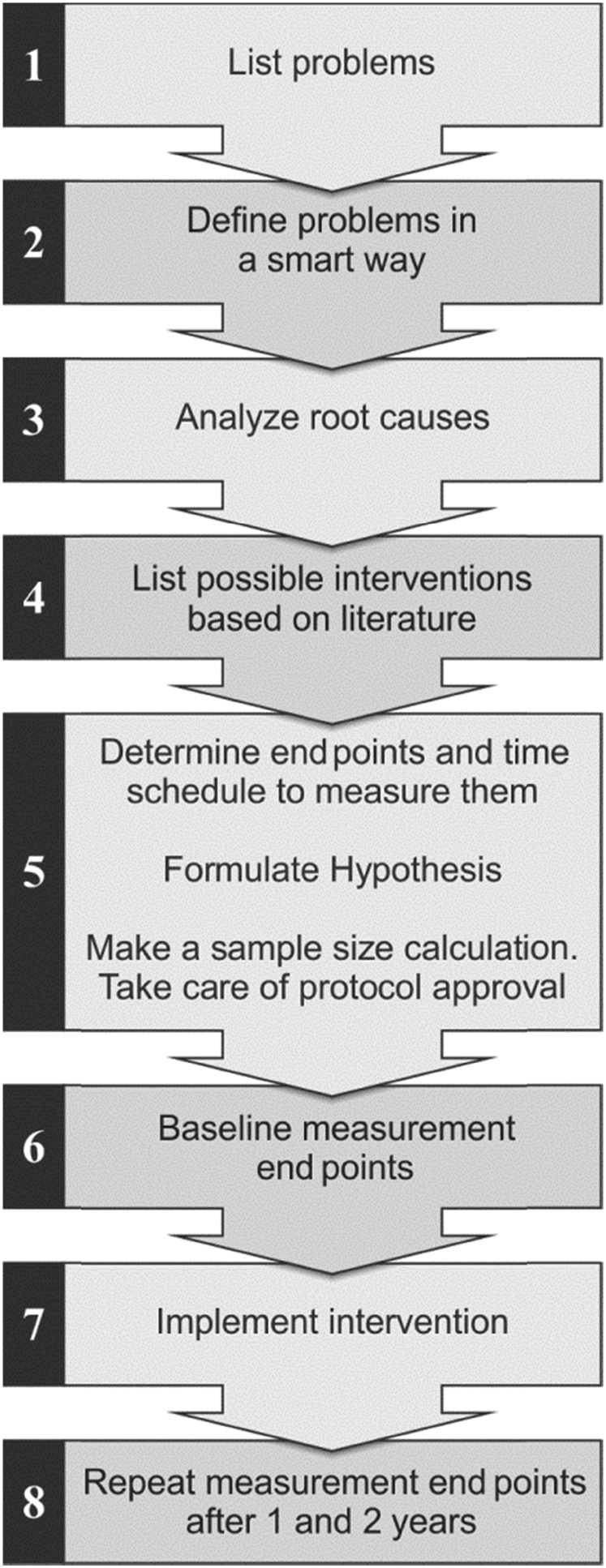
The set-up of managerial interventions.

To the best of our knowledge, reorganizations set up similarly to a prospective clinical trial are scarce in general and have not been previously attempted in radiotherapy. Although there are limitations, this design gives employees more transparency about problems, root causes interventions and effects. This approach is also well accepted by medical professionals, as it became clear from the qualitative evaluation sessions. The set-up fits our company strategy to make data-driven decisions. This approach is standard in the field of research,^43^ but this is the first attempt to do the same in the field of management. Future important managerial interventions will be taken in a similar way, according to the flowchart in Figure 6. The approach in this flowchart is “transplantable” to all other radiotherapy centres or even clinical departments, regardless of whether they are academic, peripheral or categorical.

## LIMITATIONS

First, as described, we identified two major confounding events that occurred over 1 year. These events certainly affected the formulated end points.

Second, we cannot be completely sure that the end points were purely determined by our organizational design interventions. The study's setting did not allow for a control group. So, for example, it is not unthinkable that job satisfaction increased because employees felt more comfortable with their new supervisors.

Third, we know from the literature^16^ that organizational development requires an integrated approach to structure, culture, systems, strategy and leadership. This study was confined to only structure. In 2012, we started this integrated approach, paying much more attention to culture. Further research is necessary to study the interaction between all these parameters and end points.

Finally, the findings reported here are confined to one radiotherapy centre, which may affect the generalization of the results.

## CONCLUSIONS

We initiated a hypothesis-driven reorganization trajectory of our department based on the model of prospective clinical trial. If we make a strict “intent to treat” analysis based on the main end points, this study is positive for patient care and neutral for research. The interfering events make it more difficult to establish a causal relationship between the intervention and the end points. We concluded that starting up a reorganization trajectory in a department based on the model of prospective clinical trials is a transparent approach, which is important for giving medical professionals confidence in carrying out changes in their daily practice. A systematic data-driven approach as performed in this study gives the best possible insights into the relationship between managerial interventions, expectable results and confounding events. Although the relevance of the reorganization process itself depends on the context and the country, the approach that mimics the methodology of a prospective clinical trial is transplantable to all clinical departments.

We decided to move forwards with other organizational changes based on the same model. In future research, we will take further steps in our organizational development trajectory in order to change from a function-based to a more process-based organization, and to successfully use an ambidextrous strategy for daily practice, research and innovation implementation.
